# Body proportions in patients with Turner syndrome on growth hormone treatment

**DOI:** 10.55730/1300-0144.5612

**Published:** 2022-07-28

**Authors:** Aslı Derya KARDELEN, Mine ŞÜKÜR, Esin Karakılıç ÖZTURAN, Ayşe Pınar ÖZTÜRK, Şükran POYRAZOĞLU, Firdevs BAŞ, Feyza DARENDELİLER

**Affiliations:** 1 Department of Pediatric Endocrinology, Istanbul Faculty of Medicine, Istanbul University, İstanbul, Turkey; 2 Department of Pediatric Endocrinology, Child Health Institute, Istanbul University, İstanbul, Turkey

**Keywords:** Anthropometry, auxology, body proportions, karyotype, Turner syndrome

## Abstract

**Background:**

In this cross-sectional study, we aimed to evaluate auxological measurements and detailed body proportions of recombinant human growth hormone (GH)-treated patients with Turner syndrome (TS) and compare them with a group of healthy females.

**Materials and methods:**

We evaluated 42 patients with TS who received GH treatment and 20 healthy controls. Anthropometric measurements were taken and target height, body mass index (BMI), arm span-height difference, extremity-to-trunk ratio, and Manouvrier’s skelic index were calculated.

**Results:**

The median (min–max) age of the patients at the time of evaluation was 13.6 (4.3–20.7) years, and the control group was 12.9 (3.8–23.7) years. Height, sitting height, and arm span of TS patients were significantly lower than those of the control group. Sitting height/height ratio (SHR) was in normal ranges in both groups and BMI was significantly higher in TS patients when compared to the control group. According to Manouvrier’s skelic index, TS patients had shorter legs than the control group (p = 0.001). The extremity-trunk ratio was significantly decreased in TS patients compared to healthy controls (p < 0.001). There was no significant difference between the karyotype groups in terms of these indexes.

**Conclusion:**

TS patients had short stature, increased BMI and waist circumference, normal head circumference, and decreased extremity-trunk ratio. Sitting height and leg length were short; however, the SHR standard deviation score (SDS) was in the normal range. Despite being treated with GH, TS patients had disproportionate short stature. The disproportion in TS patients was similar to short-stature homeobox-containing gene (SHOX) deficiency, which is considered to be SHOX haploinsufficiency in the etiopathogenesis of short stature.

## 1. Introduction

Turner syndrome (TS) is a chromosomal disorder caused by complete or partial X chromosome monosomy and affects approximately 1 in 2500 live-born females. TS can manifest with various clinical features depending on the karyotype and the genetic background of the affected individuals. In particular, short stature is a common finding of TS and adult height is approximately 20 cm shorter than the population average [1,2].

In a recent multicenter study, the growth curves of Turkish children with TS were created [3]. According to these data, the final height in TS without treatment is 141.9 ± 6.9 cm [4], slightly lower than the European patients [2].

Although GH secretion is normal, the reasons for short stature have been reported as low levels of free insulin-like growth factor 1 (IGF-1), increased IGF-binding protein 3 (IGFBP-3) proteolysis, IGF-1 resistance, and estradiol deficiency. However, it is thought that the absence of a copy of the short-stature homeobox-containing gene (SHOX) located in the distal pseudoautosomal region of the X chromosome mainly causes short stature [1,2]. Recombinant GH treatment in the affected children has been used since the 1990s and is now a routine part of the treatment [5].

Short stature in TS is disproportionate. The general appearance of body proportions in girls with TS is short stature with short legs and reduced arm length and they have stocky bodybuilding when compared to healthy controls [6–8]. On the other hand, few studies have investigated detailed anthropometric measurements of TS patients on GH treatment; therefore, we planned this study.

In this study, we aimed to evaluate the anthropometric features and detailed body proportions of girls with TS who received GH treatment and the effect of karyotype on these parameters. We aimed to determine the characteristics for detailed auxological findings to use for early diagnosis of girls with TS.

## 2. Materials and methods

### 2.1. Patients and data collection

Forty-two female patients with a diagnosis of TS confirmed by lymphocyte chromosomal analysis with a minimum count of 50 metaphases were enrolled in this study. All TS patients received GH treatment at a daily dose of 0.045 mg/kg and for a median of 3.7 (0.6–9.2) years.

The control group consisted of 20 healthy individuals who did not have a history of medication use. The study protocol was approved by the Ethics Committee and registered under the number 2020/1175.

In this cross-sectional study, age, birth weight and height, gestational age, and karyotypes were recorded during the evaluation. All patients underwent anthropometric measurements. Standing and sitting heights were measured using a Harpenden stadiometer (Holtain Ltd., Crymych, UK), with calibrated stadiometers, and a sitting height table. A number of anthropometric measurements were assessed to quantify possible disproportion: weight, height, head circumference, sitting height, left lower and upper leg and foot length, subischial leg length, left upper arm and forearm, hand, arm span length, biacromial length, and chest, waist, and hip circumference were taken. All measurements were performed by the same observer. Body mass index (BMI), sitting height/height ratio (SHR), arm span-height difference, extremity-to-trunk ratio [(leg length + arm span)/sitting height], Manouvrier’s skelic index [(leg length × (height-sitting height)/sitting height) × 100] were calculated. Standard deviation score (SDS) calculations were done according to the national data of the measurements [9,10]. In addition, parental height was measured and target height SDS was calculated.

For evaluating the extremity-trunk ratio Binder et al. prepared a mathematical approximation according to their data in which the extremity-trunk ratio less than 1.95 + ½ height (m) has a sensitivity of 100% and a specificity of 85% for testing SHOX haploinsufficiency [11]. A low extremity-trunk ratio indicates disproportionately short extremities. According to the Manouvrier’s skelic index, it was classified as brachiskelic (short leg) (≤87.92%), mesaskelic (normal leg) (87.93%–92.06%), and macroskelic (long leg) (≥92.07%).

### 2.2. Statistical analysis

SPSS for Windows v. 21.0 was used for statistical analysis. The results were reported as median (min–max) or as number or percentages, where appropriate. Continuous variables without normal distribution were compared using a Mann–Whitney U test for the nonparametric analysis and categorical variables using a Chi-square test in the two-group comparisons (TS and control groups). Correlations of anthropometric measures were examined using the Pearson correlation test and linear regression test in the groups. p value < 0.05 was accepted as statistically significant.

## 3. Results

The median age of the girls with TS was 13.6 years (min–max, 4.3–20.7 years old) with no difference compared to the control group. Regarding karyotype analysis, chromosomal constitution of TS patients showed 45, X in 45.2% (n = 19), mosaicism in 40.5% (n = 17), and structural aberration (46, XX, Xp/Xq) in 14.3% (n = 6) of the patients.

Nineteen percent of the girls with TS were born small for gestational age (SGA) and all of the girls in the control group were born appropriate for gestational age (AGA). Except for two patients in the control group, there was no preterm birth history in the rest of the study population. Birth weight and the gestational week did not differ between TS and control groups (p = 0.602, p = 0.182 respectively).

The median height SDS in TS patients was significantly lower than the control group (p < 0.001). Median weight SDS and head circumference SDS were in normal ranges in TS patients. BMI SDS was significantly higher in the TS patients when compared to the control group (p = 0.041). Of the TS patients, 16.6% were obese and 28.6% were overweight.

Sitting height of the TS patients was decreased when compared to the controls (p < 0.001); however, SHR was normal in both groups. The anthropometric data are tabulated in Table 1.

When TS patients were compared to their healthy controls on the basis of comprehensive measurements; Manouvrier’s skelic index was significantly different than the control group (p = 0.001). According to Manouvrier’s skelic index, the TS patients had significantly shorter legs than the control group.

Extremity to trunk ratio was significantly (p < 0.001) decreased in the TS patients compared to healthy controls. Extremity-trunk ratio was normal in 86.8% and low in %13.2 of the TS patients. All patients with low extremity-trunk ratio were over 110 cm in height. The extremity-trunk ratio was normal in the entire control group (p = 0.002).

Some of the karyotype groups of TS patients were too small for evaluation, but when comparing karyotype 45, X with the rest of the karyotypes, there was no statistical difference with respect to anthropometric measurements (Table 2).

Twenty girls with TS attained final height (growth velocity < 0.5 cm/year, bone age ≥ 14 years) and we presented their longitudinal data for the anthropometric measurement in cm in Table 3.

In the TS group, there were strong relations between height SDS (dependent variable) and arm span SDS (independent variable) (R^2^ = 0.765, p = 0.000), height SDS (dependent variable) and sitting height SDS (independent variable) (R^2^ = 0.0419, p = 0.000). There was no relationship between height SDS and SH/height ratio (R^2^ = 0.022, p = 0.347).

**Figure 1:**
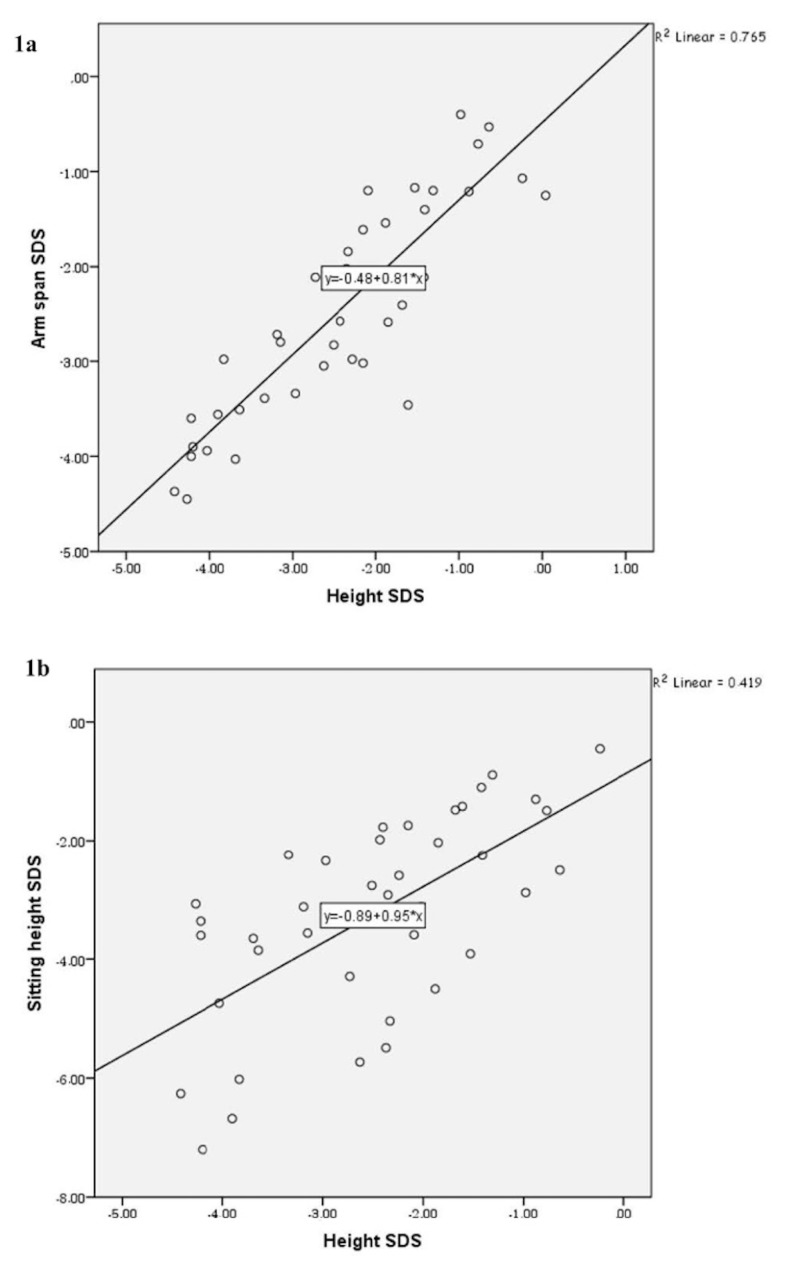
a: Linear correlation between height SDS and arm span SDS, b: Linear correlation between height SDS and sitting height SDS in the Turner syndrome group.

In the control group, there were correlations between height SDS and arm span SDS (r = 0.776, p = 0.000), height SDS and sitting height SDS (r = 0.605, p = 0.006), but there was a negative correlation between height and SH/Height ratio (r = −0.492, p = 0.028). These relationships in the TS group are shown in Figures 1a and 1b.

## 4. Discussion

Although many reports have pointed out that girls with TS have short stature, few studies have been reported on detailed anthropometric measurements. Therefore, in this study, we presented detailed anthropometric measurements of TS patients on GH treatment. The distribution of karyotype in our cohort revealed the most common karyotype as 45, X followed by mosaicism which was compatible with previous reports on karyotype diversity [12]. All TS patients were born at term, and preterm birth was not common in our study. One in five TS newborns was born SGA, which indicates that in most patients, the growth deceleration had begun during pregnancy. This was compatible with the results of Sari et al. [13].

Short stature in TS patients was reported to be because of markedly shortened lower limbs [7]. Short legs constitute the disproportion; however, the legs are reported to be disproportionate as well, with relatively short upper legs [8]. Therefore, the degree of abnormality in lower segment is a major determinant of stature in TS [7]. Baldin et al. found anthropometric measurements including height, sitting height, leg length, arm span, hand, foot, and biacromial and biiliac diameter in the TS patients were lower than those of the healthy controls except for the head circumference. Therefore, in women with short stature and normal head circumference, they suggest looking for TS [14,15]. This finding was similar to our data. The disproportion is distorted in native TS patients who are not treated with GH [14,15]. When compared to our patients with the data of native TS patients [14,15], GH-treated TS patients had disproportion too and we conclude that GH does not improve the height disproportion in TS. Height, sitting height, arm span of patients with TS on GH treatment were decreased when compared to the controls; however, head circumference was in normal ranges in both groups. Head circumference often correlates with weight and height SDS [16]. Consequently, for girls with short stature and normal head circumference (disproportional to height), we suggest looking for TS as Baldin et al.

One of the reasons for short stature in TS is SHOX haploinsufficiency. The most reliable clinical indicator of SHOX deficiency is the mesomelic shortening of the extremities compared with trunk; therefore, evaluation of SHOX patients requires measurement of arm span and sitting height as well as the standing height and leg length. Arm span is significantly reduced in comparison to standing height, and leg length is significantly shorter than the sitting height. Reductions in the ratios of arm span and forearm length to height and an increase in SHR are evidence of limb shortening [17,18]. Malaquias et al. reported that SHR SDS of the SHOX-deficient patients was 3.7 ± 1.6 and that of the TS patients was 1.9 ± 1.6; and sitting height SDS of SHOX-deficient patients was −0.9 ± 0.9 and TS patients −2.4 ± 1.4. Disproportionate short stature in patients with SHOX defects was found to be more common than TS patients [19]. In our cohort, arm spans SDS of the TS patients were lower than their height SDS. Arm span-height SDS difference was significantly low compared to healthy controls. In addition, sitting height and standing height of TS patients were low; however, SHR was normal. These findings are the evidence of the effects of SHOX haploinsufficiency in TS patients.

**Figure 2:**
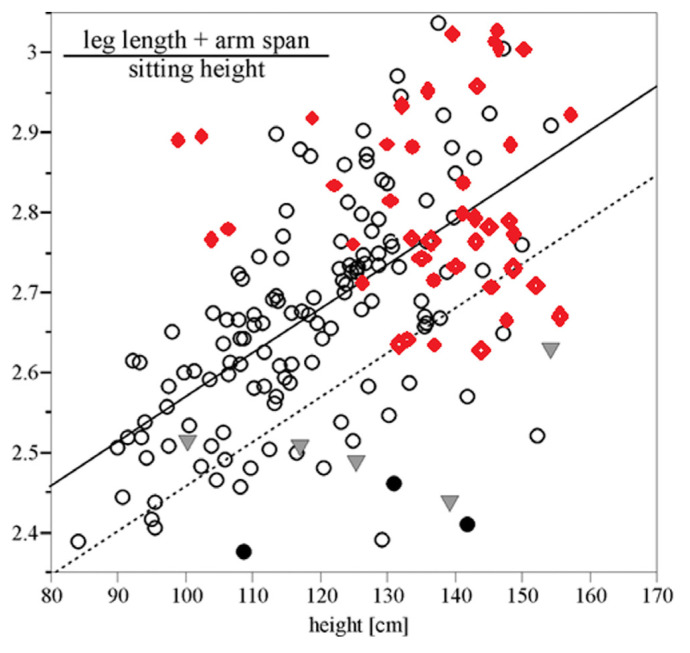
The extremity-trunk ratio of patients with Turner syndrome in the graphic created by Binder et al. The solid line indicates the mean extremities-trunk ratio for height in patients with SHOX haploinsufficiency; the lower dotted line indicates the mean extremities-trunk ratio minus 1 SD (reference 11). ♦ indicates TS patients in our study.

Binder et al. reported that SHOX haploinsufficiency can be estimated to be 12% in short children with extremity-trunk ratio less than 1.95 + 1/2 height (m). More interestingly, a normal or high extremity-trunk ratio excluded SHOX haploinsufficiency in children with a height above 110 cm. In children shorter than 110 cm, this test has a lower sensitivity and specificity and should be used with caution. SHOX mutation screening was suggested to be restricted to short school-age children with an extremity-trunk ratio less than 1.95 + 1/2 height (m) [11]. Wolters et al. reported that a decreased extremity-trunk ratio has a sensitivity of 59% and specificity of 91% to estimate SHOX deficiency [20]. To our knowledge, our study is the first study investigating extremity trunk ratio in patients with TS and we found that 13.2% of TS patients have low extremity-trunk ratio; however, it was normal in the entire control group. We marked the extremity-trunk ratio of our TS patients in the graphic created by Binder et al. (Figure 2) [11].

Furthermore, in our study, there is no statistical difference between the karyotype 45, X and the remaining karyotypes on any of the variables measured and this was compatible with the results of Gravholt et al. [21]. Some of the karyotype subgroups in the study performed by Gravholt et al. were too small for evaluation; therefore, they compared the karyotype 45, X with the rest of the karyotypes as in our study, and anthropometric proportions showed no statistical difference between groups [21].

There are a very small number of studies which evaluate detailed measurements of TS patients who attained final height. Baldin and Gravholt declared the measurements of adult TS patients in centimeters; therefore, we presented the results of these studies and the TS patients who attained the final height in our study in Table 3 [14,15,21]. According to these data, body proportions including BMI were similar to the other studies, while waist circumference was higher than in other studies. This was the result of evident visceral adiposity of our patients.

Differences in age at the start of GH and the duration of treatment were the limitations of this study. However, it was shown that GH did not modify the body proportions, including sitting height, leg length, and SHR. When the nontreated and GH-treated TS patients were compared for standing and sitting height; SHR, leg, hand, and foot lengths were found to be higher in the GH-treated patients [14]. All patients with TS (treated or not treated with GH) showed lower values of sitting height, leg length, and high SHR when compared to healthy controls [15]. However, in another study, the increase in height after long-term GH treatment is accompanied by an even greater increase in the size of the feet and a moderate improvement of the disproportion between height and sitting height [22]. Additionally, shape values of sitting height had decreased to normal values, those of foot had increased, and both remained constant after GH discontinuation. Hand measurement and biiliac and biacromial measurements did not change significantly [23]. The other limitation is the relatively small number of patients, which is a result of the fact that this was a single-center study for an uncommon disease.

## 5. Conclusion

Data on body proportions of TS patients are scarce and mostly focused on final height. Girls with short stature and normal head circumference (disproportional to height), it is important to look for TS. Despite being treated with GH, body disproportion persisted when compared to reported data of nontreated TS patients [14,15]. The disproportion in TS patients was similar to SHOX deficiency. The anthropometric parameters used in the diagnosis of SHOX deficiency could be used in the diagnosis of TS patients. A low extremity-trunk ratio, which is easily measured, may be used as a predictor of TS patients. In particular, TS patients should be followed up with larger series in this area and longitudinal studies are needed on this issue.

## Figures and Tables

**Table 1:** Anthropometric measurements of patients with Turner syndrome on GH treatment and healthy controls [median (min–max)].

	Turner syndrome (n = 42)	Control grup (n = 20)	p

**Age (years)**	13.6 (4.3 to 20.7)	12.9 (3.8 to 23.7)	0.150

**Birth weight SDS**	−1.1 (−3.4 to 1.1)	−0.6 (−4.3 to 2.8)	0.602

**Birth height SDS**	0.7 (−3.0 to 0.8)	0.3 (−1.1 to 1.2)	0.022

**Paternal height SDS**	−0.9 (−3.1 to 2.2)	−0.5 (−3.0 to 0.6)	0.672

**Maternal height SDS**	−0.8 (−3.7 to 1.6)	−0.6 (−2.8 to 1.2)	0.503

**Target height SDS**	−0.9 (−2.3 to 0.9)	−0.5 (−2.1 to 0.6)	0.707

**Weight SDS**	−0.7 (−5.5 to 3.7)	0.0 (−2.6 to 1.4)	0.180

**Height SDS**	−2.3 (−4.4 to 0.04)	−0.1 (−1.5 to 1.2)	**<0.001**

**BMI SDS**	0.8 (−3.1 to 3.7)	0.0 (−2.6 to 1.4)	**0.041**

**Head circ SDS**	−1.1 (−4.3 to 1.8)	−0.7 (−2.3 to 0.6)	0.228

**SH SDS**	−3.1 (−7.2 to −0.5)	−1.1 (−2.7 to 0.8)	**<0.001**

**SHR**	0.54 (0.50 to 0.56)	0.53 (0.53 to 0.57)	0.593

**SHR SDS**	0.3 (−1.5 to 1.8)	0.05 (−2.3 to 1.6)	0.310

**Arm span SDS**	−2.5 (−4.5 to −0.4)	0.4 (−1.4 to 1.9)	**<0.001**

**Arm span–Height SDS difference**	0.2 (−3.1 to 4.4)	0.8 (−3.6 to 2.6)	**0.026**

**Waist SDS**	1.2 (−1.9 to 3.5)	0.9(−2.1 to 2.8)	0.447

**Manouvrier’s skelic index %**	95 (79.3 to 120)	112.3 (98.5 to 124.3)	**<0.001**

**Manouvrier’s skelic index n(%)**			
**Brachiskelic**	17 (40.5%)	0	
**Mesaskelic**	4 (9.5%)	0	**0.001**
**Macroskelic**	21 (50%)	20 (100%)	

**Extremity–trunk ratio**	2.8 (2.6 to 3.2)	3.0 (2.8 to 3.2)	**<0.001**

**Extremity–trunk ratio n(%)**			
**Low**	5 (13.2)	–	
**Normal**	13 (34.2)	–	**0.002**
**High**	20 (52.6)	20 (100)	

SDS, Standard deviation score; BMI, body mass index; SH, sitting height; SHR, sitting height ratio

**Table 2:** Comparison of anthropometric measurements of theTurner syndrome patients with 45, X karyotype with other karyotypes [median (min–max)].

	45, X (n=19)	Non 45, X (n=23)	p
**Age (years)**	14 (5.0 to 20.7)	13.3 (4.3 to 20.1)	0.528
**Birth weight SDS**	−0.8 (−3.2 to 0.8)	−1.2 (−3.4 to 1.0)	0.349
**Birth height SDS**	−0.7 (−3.0 to 0.8)	−0.7 (−2.5 to 0.3)	0.756
**Target height SDS**	−0.8 (−2.3 to 0.5)	−0.9 (−2.1 to 0.9)	0.527
**Weight SDS**	−0.4 (−4.9 to 1.5)	−0.8 (−5.5 to 3.7)	0.370
**Height SDS**	−2.2 (−4.2 to −0.24)	−2.4 (−4.4 to 0.04)	0.503
**BMI SDS**	1.1 (−2.7 to 3.3)	0.3 (−3.1 to 3.7)	0.350
**Head circumference SDS**	−0.9 (−3.9 to 1.3)	−1.4 (−4.3 to 1.8)	0.161
**SH SDS**	−3.0 (−7.2 to −0.5)	−1.1 (−2.7 to 0.8)	0.835
**SHR**	0.54 (0.50 to 0.56)	0.53 (0.53 to 0.57)	0.824
**SHR SDS**	0.2 (−1.5 to 1.8)	0.4 (−0.9 to 1.8)	0.601
**Arm span SDS**	−2.2 (−4.0 to −0.4)	−2.8 (−4.5 to −0.5)	0.553
**Arm span–Height SDS difference**	0.2 (−2.8 to 3.6)	0.3 (−3.1 to 4.4)	0.424
**Waist SDS**	1.4 (−1.8 to 3.0)	1.1(−1.9 to 3.5)	0.419
**Manouvrier’s skelic index**	95.8 (81.6 to 115.2)	94.1 (79.3 to 119.9)	0.909
**Extremity–trunk ratio**	2.8 (2.6 to 3.2)	2.8 (2.6 to 3.2)	0.990

SDS, standard deviation score; BMI, body mass index; SH, sitting height; SHR, sitting height ratio.

**Table 3:** Anthropometric measurements of TS patients who attained final height Mean **±** SD (95% confidence limits).

Reference no	[21]* n=79	[13, 14]* n=52	[13, 14]** n=30	This study** n=20
**Weight (kg)**	56.4 ± 12.7 (53.5–59.2)	48.4 ± 10.4 (45.5–51.3)	49.7 ± 10.6 (44.7–54.6)	54.2 ± 15.4 (48.1–62.2)
**Height (cm)**	146.8 ± 6.6 (145.3–148.3)	143.9 ± 5.0 (142.5–145.5)	146.2 ± 4.1 (144.7–147.8)	146.5 ± 7.1 (143.2–150)
**BMI (kg/m** **^2^** **)**	26.1 ± 5.0 (25.0 ± 27.2)	23.2 ± 4.7 (21.9–24.6)	24.3 ± 4.6 (22.2–26.4)	25.1 ± 6.1 (22.7–28.2)
**Head circ (cm)**	55.2 ± 2.0 (54.8–55.7)	54.5 ± 1.9 (54.0–55.0)	54.4 ± 1.7 (53.7–55.1)	53.9 ± 1.5 (53.3–54.7)
**Upper arm (cm)**	ND	ND	ND	23.8 ± 4.4 (21.8–26.0)
**Forearm (cm)**	ND	ND	ND	21.5 ± 2.7 (20.3–22.8)
**Lower leg length (cm)**	ND	ND	ND	35.6 ± 5.5 (33.3–38.4)
**Upper leg length (cm)**	ND	ND	ND	36.8 ± 5.9 (34.3–39.8)
**Leg length (cm)** #	68.2 ± 4.4 (67.2–69.1)	65.6 ± 3.9 (64.5–66.7)	66.3 ± 2.8 (64.7–67.1)	69.0 ± 4.3 (66.9–71.0)
**Sitting height (cm)**	78.6 ± 3.6 (77.8–79.4)	78.3 ± 3.6 (77.3–79.3)	78.3–4.1 (76.3–80.2)	77.6 ± 3.6 (75.9–79.4)
**SHR**	0.54 ± 0.02 (0.53–0.54)	0.54 ± 1.5 (0.54–0.55)	0.54 ± 1.5 (0.53–0.55)	0.53 ± 0.01 (0.53–0.54)
**Waist (cm)**	76.4 ± 11.4 (73.9–79)	77.8 ± 11.3 (74.7–80.9)	75.2 ± 8.9 (71.0–79.4)	83.1 ± 12.2 (78.0–89.0)
**Hip (cm)**	89.7 ± 7.8 (88.0–91.5)	88.9 ± 8.8 (86.5–91.4)	87.9 ± 8.6 (83.9–92.0)	86 ± 13 (80.7–93.0)
**Hand (cm)**	17.0 ± 1.1 (6.7–17.2)	17.2 ± 1.5 (16.7–17.6)	17.9 ± 0.8 (17.4–18.3)	13.5 ± 2.3 (12.4–14.6)
**Foot (cm)**	22.4 ± 1.2 (22.2–22.7)	21.6 ± 1.8 (21.1–22.2)	21.7 ± 1.6 (21.2–22.9)	17.4 ± 1.9 (16.5–18.3)
**Arm span (cm)**	147.7 ± 7.0 (146.1–149.3)	144.9 ± 9.0 (142.4–147.4)	144.2 ± 8.0 (141.8–146.9)	148.4 ± 6.8 (145.6–151.9)
**Biacromial diameter (cm)**	36.5 ± 1.9 (36.1–36.9)	34.0 ± 2.4 (33.3–34.7)	34.0 ± 1.5 (33.4–34.5)	33.5 ± 3.3 (32.1–35.2)
**Chest (cm)**	ND	ND	ND	68 ± 8.6 (63.3–67.6)

* Patients who did not receive GH treatment,

** Patients who received GH treatment,

# Leg length was calculated as the difference between standing and sitting height. In addition subischial leg length of the TS patients who attained final height was 72.4 ± 11.4 (67.7–78.1) cm.

BMI, body mass index; ND, not determined; SHR, sitting height ratio

## References

[1] Sybert VP McCauley E Turner’s syndrome The New England Journal of Medicine 2004 351 12 1227 1238 10.1056/NEJMra030360 15371580

[2] Lyon AJ Preece MA Grant DB Growth curve for girls with Turner syndrome Archives of Disease in Childhood 1985 60 10 932 935 10.1136/adc.60.10.932 4062345 PMC1777486

[3] Darendeliler F Yeşilkaya E Bereket A Baş F Bundak R Growth curves for Turkish girls with Turner syndrome: Results of the Turkish Turner syndrome study group Journal of Clinical Research in Pediatric Endocrinology 2015 7 3 183 191 10.4274/jcrpe.2023 26831551 PMC4677552

[4] Bereket A Turan S Elçioğlu N Hacihanefioğlu S Memioğlu N Adult height in Turkish patients with Turner syndrome without growth hormone treatment The Turkish Journal of Pediatrics 2008 50 5 415 417 19102043

[5] Saenger P Wikland KA Conway GS Davenport M Gravholt CH Recommendations for the diagnosis and management of Turner syndrome The Journal of Clinical Endocrinology and Metabolism 2001 86 7 3061 3069 10.1210/jcem.86.7.7683 11443168

[6] Ikeda Y Higurashi M Egi S Ohzeki N Hoshina H An anthropometric study of girls with the Ullrich-Turner syndrome American Journal of Medical Genetics 1982 12 3 271 280 10.1002/ajmg.1320120305 7114090

[7] Neufeld ND Lippe BM Kaplan SA Disproportionate growth of the lower extremities. A major determinant of short stature in Turner’s syndrome American Journal of Diseases of Children 1978 132 3 296 298 10.1001/archpedi.1978.02120280080018 629248

[8] Rongen-Westerlaken C Rikken B Vastrick P Jeuken AH de Lange MY Body proportions in individuals with Turner syndrome. The Dutch Growth Hormone Working Group European Journal of Pediatrics 1993 152 10 813 817 10.1007/BF02073377 8223783

[9] Neyzi O Bundak R Gökçay G Günöz H Furman A Reference values for weight, height, head circumference, and body mass index in Turkish children Journal of Clinical Research in Pediatric Endocrinology 2015 7 4 280 293 10.4274/jcrpe.2183 26777039 PMC4805217

[10] Demir K Konakçı E Özkaya G Kasap Demir B Özen S New features for child metrics: Further growth references and blood pressure calculations Journal of Clinical Research in Pediatric Endocrinology 2020 12 2 125 129 10.4274/jcrpe.galenos.2019.2019.0127 31475511 PMC7291402

[11] Binder G Ranke MB Martin DD Auxology is a valuable instrument for the clinical diagnosis of SHOX haploinsufficiency in school-age children with unexplained short stature The Journal of Clinical Endocrinology and Metabolism 2003 88 10 4891 4896 10.1210/jc.2003-030136 14557470

[12] Elsheikh M Dunger DB Conway GS Wass JA Turner’s syndrome in adulthood Endocrine Reviews 2002 23 1 120 140 10.1210/edrv.23.1.0457 11844747

[13] Sari E Bereket A Yeşilkaya E Baş F Bundak R Anthropometric findings from birth to adulthood and their relation with karyotpye distribution in Turkish girls with Turner syndrome American journal of Medical Genetics 2016 170A 4 942 948 10.1002/ajmg.a.37498 26788866

[14] Baldin AD Fabbri T Siviero-Miachon AA Spinola-Castro AM Lemos-Marini SH Effects of growth hormone on body proportions in Turner syndrome compared with non-treated patients and normal women Journal of Endocrinological Investigation 2010 33 10 691 695 10.1007/BF03346671 20354352

[15] Baldin AD Fabbri T Siviero-Miachon AA Spinola-Castro AM de Lemos-Marini SH Growth hormone effect on body composition in Turner syndrome Endocrine 2011 40 3 486 491 10.1007/s12020-011-9504-z 21720878

[16] Geraedts EJ van Dommelen P Caliebe J Visser R Ranke MB Association between head circumference and body size Hormone Research in Paediatrics 2011 75 3 213 219 10.1159/000321192 21311161

[17] Binder G Short stature due to SHOX deficiency: Genotype, phenotype, and therapy Hormone Research in Paediatrics 2011 75 2 81 89 10.1159/000324105 21325865

[18] Rappold G Blum WF Shavrikova EP Crowe BJ Roeth R Genotypes and phenotypes in children with short stature: clinical indicators of SHOX haploinsufficiency Journal of Medical Genetics 2007 44 5 306 313 10.1136/jmg.2006.046581 17182655 PMC2597980

[19] Malaquias AC Scalco RC Fontenele EG Costalonga EF Baldin AD The sitting height/height ratio for age in healthy and short individuals and its potential role in selecting short children for SHOX analysis Hormone Research in Paediatrics 2013 80 6 449 456 10.1159/000355411 24296787

[20] Wolters B Lass N Wunsch R Böckmann B Austrup F Short stature before puberty: Which children should be screened for SHOX deficiency? Hormone Research in Paediatrics 2013 80 4 273 280 10.1159/000354989 24051572

[21] Gravholt CH Weis Naeraa R Reference values for body proportions and body composition in adult women with Ullrich-Turner syndrome American Journal of Medical Genetics 1997 72 4 403 408 10.1002/(sici)1096-8628(19971112)72:4<403::aid-ajmg6>3.0.co;2-r 9375721

[22] Sas TC Gerver WJ de Bruin R Stijnen T de Muinck Keizer-Schrama SM Body proportions during long-term growth hormone treatment in girls with Turner syndrome participating in a randomized dose-response trial The Journal of Clinical Endocrinology and Metabolism 1999 84 12 4622 4628 10.1210/jcem.84.12.6225 10599729

[23] Bannink EM van der Palen RL Mulder PG de Muinck Keizer-Schrama SM Long-term follow-up of GH-treated girls with Turner syndrome: BMI, blood pressure, body proportions Hormone Research 2009 71 6 336 342 10.1159/000223418 19506391

